# 

**Published:** 1878-08

**Authors:** 


### CONTENTS.

### COMMUNICATIONS.

Report on Therapeutics.—*By J. T. M'Millan, D. D. S.*.................. 311
Surgical Operations on the Pulp and the best means of Extracting it.
—*By Dr. C. G. Edwards*.................................................. 317
Annual Address.—*By A. Wilkes Smith*....................................... 323

### PROCEEDINGS OF SOCIETIES.

Discussions before the Kentucky State Dental Association .............. 330
Proceedings of the Nineteenth Annual Meeting of the Northern Ohio
Dental Association.......................................................... 340
Correspondence.............................................................. 348

### EDITORIAL.

The Best Dentist's Chair.................................................. 349
Journalistic Consolidation ................................................ 351
Erratum..................................................................... 352

